# Preclinical imaging of the co-stimulatory molecules CD80 and CD86 with indium-111-labeled belatacept in atherosclerosis

**DOI:** 10.1186/s13550-015-0157-4

**Published:** 2016-01-04

**Authors:** Romana Meletta, Adrienne Müller Herde, Patrick Dennler, Eliane Fischer, Roger Schibli, Stefanie D. Krämer

**Affiliations:** Center for Radiopharmaceutical Sciences ETH-PSI-USZ, Institute of Pharmaceutical Sciences, Department of Chemistry and Applied Biosciences, ETH Zurich, Vladimir-Prelog-Weg 3/4, CH-8093 Zurich, Switzerland; Center for Radiopharmaceutical Sciences ETH-PSI-USZ, Paul Scherrer Institute, OIPA10A, 5232 Villigen-PSI, Switzerland

**Keywords:** Co-stimulatory molecules, CD80, CD86, Belatacept, Atherosclerosis, Inflammation, Imaging

## Abstract

**Background:**

The inflammatory nature of atherosclerosis provides a broad range of potential molecular targets for atherosclerosis imaging. Growing interest is focused on targets related to plaque vulnerability such as the co-stimulatory molecules CD80 and CD86. We investigated in this preclinical proof-of-concept study the applicability of the CD80/CD86-binding fusion protein belatacept as a probe for atherosclerosis imaging.

**Methods:**

Belatacept was labeled with indium-111, and the binding affinity was determined with CD80/CD86-positive Raji cells. In vivo distribution was investigated in Raji xenograft-bearing mice in single-photon emission computed tomography (SPECT)/CT scans, biodistribution, and ex vivo autoradiography studies. Ex vivo SPECT/CT experiments were performed with aortas and carotids of ApoE KO mice. Accumulation in human carotid atherosclerotic plaques was investigated by in vitro autoradiography.

**Results:**

^111^In-DOTA-belatacept was obtained in >70 % yield, >99 % radiochemical purity, and ~40 GBq/μmol specific activity. The labeled belatacept bound with high affinity to Raji cells. In vivo, ^111^In-DOTA-belatacept accumulated specifically in Raji xenografts, lymph nodes, and salivary glands. Ex vivo SPECT experiments revealed displaceable accumulation in atherosclerotic plaques of ApoE KO mice fed an atherosclerosis-promoting diet. In human plaques, binding correlated with the infiltration by immune cells and the presence of a large lipid and necrotic core.

**Conclusions:**

^111^In-DOTA-belatacept accumulates in CD80/CD86-positive tissues in vivo and in vitro rendering it a research tool for the assessment of inflammatory activity in atherosclerosis and possibly other diseases. The tracer is suitable for preclinical imaging of co-stimulatory molecules of both human and murine origin. Radiolabeled belatacept could serve as a benchmark for future CD80/CD86-specific imaging agents.

**Electronic supplementary material:**

The online version of this article (doi:10.1186/s13550-015-0157-4) contains supplementary material, which is available to authorized users.

## Background

The innate and adaptive immune systems interact in a well-orchestrated manner upon recognition of an antigen. After antigen exposure, professional antigen-presenting cells (APC), such as dendritic cells (DC), lose phagocytic action simultaneously gaining antigen-presenting capability by the upregulation of major histocompatibility complex (MHC) receptors and the co-stimulatory molecules CD80 (B7.1) and CD86 (B7.2) [1]. The interactions of CD80 and CD86 with their T cell receptor cytotoxic T lymphocyte protein 4 (CTLA-4, CD152) and CD28 belong to the best-characterized co-stimulatory pathways in immune response, determining whether antigen exposure results in immune tolerance or T cell activation [2]. The interactions between the co-stimulatory molecules and their T cell receptors and the resulting immune response are determining factors for the course of various diseases including atherosclerosis, cancer, transplant rejection, and autoimmune diseases [1, 3, 4].

In atherosclerosis research, Buono et al. demonstrated that LDL receptor (Ldlr) knockout (KO) mice lacking CD80 and CD86 displayed a delayed atherosclerosis progression compared to control Ldlr KO mice [5]. The involvement of CD80 and CD86 in vascular remodeling was presented by Ewing et al. in a femoral artery cuff mouse model [6]. We recently found significantly higher relative messenger RNA (mRNA) levels of CD80 and CD86 in human vulnerable than stable carotid plaques, supported by immunohistochemistry data [7]. Our results were in agreement with a study on human carotid and coronary plaques by Erbel et al. [8]. They observed a higher CD86 mRNA expression in human carotid plaques of symptomatic than asymptomatic patients. Their immunohistochemical analysis revealed that virtually all mature DCs were CD86-positive and in close proximity to activated T cells. Further evidence for an involvement of mature APCs in atherosclerosis was presented in an expression analysis of human carotid endarterectomized plaques [9]. CD11c, CD80, CD83, and CD86 mRNA expression was significantly higher in vulnerable than stable plaques. The increase in vulnerable plaques was more pronounced for the mature subpopulation of DCs than the total number including immature DCs [9]. Altogether, these studies indicate that advanced atheroma contain a population of fully maturated DCs and macrophages expressing co-stimulatory molecules and that these APCs could be efficient regulators of T cell activity in atherosclerosis. Antigen presentation is not restricted to lymph nodes but additionally occurs in atherosclerotic plaques as supported by co-localization data of DCs and T cells [7, 8, 10].

Since activated APCs that express CD80 and CD86 are a primary component of atherosclerotic lesions and the amount of APCs correlates with plaque vulnerability [9–11], CD80 and CD86 may be promising imaging targets in atherosclerosis. We recently evaluated CD80 as a target for non-invasive imaging by positron emission tomography (PET) [7]. An oxodihydropyrazolocinnoline derivative labeled with carbon-11 showed high binding affinity to CD80 and bound to CD80-positive Raji xenografts and in a displaceable manner to endarterectomized human carotid plaques both in vitro. However, the low tissue distribution of the carbon-11-labeled ligand was a limiting factor in pilot PET experiments with mice [7].

Abatacept (CTLA4-Ig, Orencia^®^, Bristol Myers Squibb) and belatacept (LEA29Y, Nulojix^®^, Bristol Myers Squibb) are 90-kDa fusion proteins of the extracellular domain of human CTLA-4 and a modified Fc fragment of a human IgG1, used in the therapy against rheumatoid arthritis and transplant rejection, respectively, inhibiting CD80/CD86-mediated T cell activation. Belatacept differs by two amino acids from abatacept and the CTLA-4 extracellular domain, resulting in a 10-fold increased inhibition of T cell co-stimulation in vitro [12]. Surface plasmon resonance measurements revealed high binding affinities of belatacept to human and mouse CD80 as well as CD86 [12, 13].

In the present proof-of-concept study, we evaluated the potential of CD80/CD86-specific belatacept for atherosclerosis imaging. We labeled belatacept with the gamma-emitting nuclide indium-111 (physical half-life 2.8 days) and characterized its binding affinity to human CD80/CD86 and its stability in plasma in vitro. The ^111^In-labeled probe was further evaluated by in vivo and ex vivo single-photon emission computed tomography (SPECT) with mice regarding its accumulation in CD80/CD86-positive murine tissues and human CD80/CD86-positive xenografts. Moreover, ex vivo SPECT scans were acquired in a mouse model of atherosclerosis. Finally, the in vitro binding of ^111^In-DOTA-belatacept to human carotid plaques was compared with histological markers of plaque inflammation.

## Methods

### Ethics, consent, and permissions

All procedures performed in studies involving human participants were in accordance with the ethical standards of the institutional and national research committees and with the 1964 Helsinki declaration and its later amendments or comparable ethical standards. Informed consent was obtained from all individual patients included in the study prior to surgery.

All applicable international, national, and institutional guidelines for the care and use of animals were followed, in particular the Swiss legislation and FELASA guidelines. All experiments with animals were approved by the veterinary office of the Kanton Zurich.

### Conjugation, radiolabeling, quality control, and stability

Commercially available belatacept (Nulojix^®^, Bristol Myers Squibb) was conjugated to *p*-SCN-Bn-DOTA (S-2-(4-isothiocyanatobenzyl)-1,4,7,10-tetraazacyclododecane tetraacetic acid, B205, Macrocyclics, Dallas, TX) and labeled with indium-111 according to a published procedure [14]. The conjugation reaction is described in Additional file 1. The conjugate was characterized by liquid chromatography-mass spectrometry (LC-MS).

For radiolabeling, each 10 μg protein conjugate was reacted with 4 MBq ^111^In (Mallinckrodt, Dublin, Ireland) as optimized by incubation of a fixed amount of belatacept-DOTA with increasing amounts of ^111^In. The reaction mixture for the radiolabeling contained 300–350 μg belatacept-DOTA (175 μL), 0.1 M ammonium acetate buffer (pH 6, 60 μL), and ^111^In (^111^InCl_3_ in 0.02 N HCl, 213–228 MBq, 215 μL, Mallinckrodt). After 1 h incubation at room temperature (rt), the reaction was quenched by the addition of 50 mM EDTA solution (10 %, *v*/*v*), mixed and allowed to incubate for 5 min at rt. The product was purified, and its stability in phosphate-buffered saline (PBS) and plasma was investigated as described in Additional file 1.

### In vitro experiments

Cell culture, immunocytochemistry, binding studies with Raji and NCI-H69 cells and tissue staining are described in detail in Additional file 1. In brief, Raji (human Burkitt’s lymphoma cell line, ATCC CCL-86) and NCI-H69 cells (human lung small cell carcinoma cell line, ATCC HTB-119) were purchased from the German Collection of Microorganisms and Cell Cultures (DSMZ, Braunschweig, Germany) and were cultured according to the supplier’s protocols.

For immunocytochemistry, the following primary antibodies were used: anti-CD80 1:100 (2A2, ab86473, Abcam, Cambridge, UK), anti-CD86 1:200 (EP1158Y, ab53004, Abcam, Cambridge, UK), and anti-CTLA-4 1:100 (MO6, clone 2F1, H00001493-M06, Abnova, Taipei, Taiwan). Additionally, the following secondary antibodies were used: goat anti-rabbit IgG H&L 1:1000 (FITC, ab6717, Abcam, Cambridge, UK) and goat anti-mouse IgG H&L 1:1000 (FITC, ab6785, Abcam, Cambridge, UK). Cells were fixed in paraformaldehyde and permeabilized with methanol. Nuclei were stained with 4′,6-diamidino-2-phenylindole (DAPI). Binding affinity was determined in saturation binding and Lindmo assays with Raji and NCI-H69 cells as described in Additional file 1.

### In vivo and ex vivo studies with xenograft-bearing mice

Five-week-old female CD1 nu/nu mice (Crl: CD1-*Foxn*^*1nu*^) from Charles River Laboratories (Sulzfeld, Germany) were fed a normal chow diet ad libitum. At the age of 6 weeks, these immune-deficient mice were subcutaneously inoculated in their shoulder region with 1 × 10^7^ Raji cells in 100 μL Matrigel (BD Biosciences, Oxford, UK) (4 animals both sides, 6 animals right side). NCI-H69 cells (1 × 10^7^ in 100 μL Matrigel) were subcutaneously inoculated in the shoulder region of CD1 nu/nu mice 2 weeks afterwards (2 animals both sides, 6 animals with Raji xenografts, see above, opposite side). In vivo SPECT/CT scans, ex vivo autoradiography, and biodistribution experiments were performed 2 weeks after the second inoculation according to Additional file 1: Table S1. Animals were injected intravenously with 10 MBq ^111^In-DOTA-belatacept (25 μg, baseline) or 10 MBq ^111^In-DOTA-belatacept and 500 μg unlabeled belatacept (blockade). In vivo and ex vivo experiments were performed 48 h after tracer injection or as indicated.

### ApoE KO atherosclerosis mouse models and C57BL/6 control mice

Eleven-week-old male C57BL/6 mice (control) and four-week-old male ApoE KO mice (B6.129P2-Apoe^tm1Unc/J^) were obtained from Charles River Laboratories. Two C57BL/6 mice and two ApoE KO mice were fed a normal chow diet ad libitum. All other ApoE KO mice (*n* = 4) received a modified Western-type diet containing 21 % fat, 0.25 % cholesterol, and 19.5 % casein (Kliba Nafag, Kaiseraugst, Switzerland) ad libitum. In two ApoE KO animals, fed a modified Western-type diet for 4 weeks, flow-modifying implants (cuff) were placed around the right and left common carotid arteries according to previous publications, inducing atherosclerotic plaque formation up- and downstream [15, 16]. A detailed characterization of the mouse model under the above conditions, including a histological assessment, gene expression analysis, and oil red o staining, is shown elsewhere (Meletta et al., manuscript submitted). Mice were used for ex vivo SPECT/CT experiments 19 weeks after surgery. ^111^In-DOTA-belatacept (10 MBq, 25 μg, baseline) or 10 MBq ^111^In-DOTA-belatacept and 500 μg unlabeled belatacept (blockade) was injected into the tail vein of the animals. Mice were euthanized 48 h after tracer injection for ex vivo SPECT/CT imaging and oil red o staining of the excised aorta and carotids. The two mice with implants were in addition scanned before euthanasia.

### SPECT/CT imaging

In vivo and ex vivo SPECT/CT scans were conducted with a four-head multiplexing multipinhole camera (NanoSPECT/CT, Bioscan, Washington, DC, USA). The acquisition time per view depended on the amount of radioactivity at scan start in the field of view, and the resulting scan times ranged from 20 min to 1 h (in vivo scans) and 14.5 to 15.5 h (ex vivo scans). In vivo scans were performed 48 h after tracer injection or as indicated. For ex vivo studies, animals were euthanized ∼48 h after tracer injection and SPECT/CT images were acquired after sample preparation. For in vivo SPECT/CT scans, animals were anesthetized with isoflurane (1.5–4 %) in oxygen. SPECT data were reconstructed with HiSPECT software (Scivis GmbH, Göttingen, Germany), and images were generated with the Nucline Software (Bioscan). The fused SPECT and CT data were analyzed using VivoQuant image post-processing software (inviCRO Imaging Services and Softwares, Boston, USA) and PMOD biomedical image quantification software (PMOD Technologies, Zurich, Switzerland). For the ex vivo scans, volumes of interest (VOIs, ~1 cm^3^) were drawn manually to include aorta and carotids. A VOI of equal size was drawn outside the tissue samples to define background radioactivity in the absence of tissue. Tissue-to-background and tissue baseline-to-blocked ratios were calculated from the averaged 5 voxels with the highest radioactivity per VOI.

### Biodistribution experiments

Post-mortem biodistribution studies with inoculated CD1 nu/nu mice (Raji xenograft right and NCI-H69 xenograft left shoulder) were performed with three baseline animals and three blockade animals. Animals were euthanized with CO_2_ followed by cervical dislocation. Tissues, organs, and a defined volume of original injectate were weighed and counted for radioactivity in a gamma counter (Packard Cobra II Auto Gamma, PerkinElmer). Accumulated tissue radioactivity was expressed as % injected dose (Bq) per g tissue (% ID/g).

### Ex vivo autoradiography

For ex vivo autoradiographic studies, CD1 nu/nu mice bearing either Raji or NCI-H69 xenografts on both shoulders were injected with ~10 MBq tracer and euthanized after 48 h as described above. Tumors were dissected, embedded in Tissue-Tek O.C.T. medium, and frozen at −80 °C, and sections of 5 μm were prepared using a cryostat. After drying, frozen sections were exposed to super-resolution imager plates (PerkinElmer, Waltham, MA, USA) for 5 min and analyzed by a Cyclone^®^ Plus Storage Phosphor System (PerkinElmer). Radioactivity signals were quantified with the software OptiQuant (5.0, PerkinElmer).

### Human atherosclerotic carotid plaques

Atherosclerotic carotid plaques were obtained from patients undergoing carotid endarterectomy (CEA) at the University Hospital of Zurich, Switzerland. Indications for CEA were stroke, transient ischemic attack, on-pump surgery to prevent perioperative stroke, ultrasound diagnosis revealing a soft and unstable plaque, or other surgical interventions (e.g. aortic valve, coronary artery surgery). Twenty-three patients were included in this study with an average age of 75.4 ± 6.8 years, 16 were of male gender, 4 females, and 3 were unknown. Plaque material was collected from all patients from the common, external, and internal carotid artery. Available details of the patients and the samples used in this study are shown in Additional file 1: Table S2. Plaque specimens were stored in RNAlater^®^ solution (Sigma-Aldrich, St. Louis, USA) at −80 °C. After thawing, plaques were embedded in Tissue-Tek O.C.T. medium and cryosections (20 μm) were prepared with a cryostat. Frozen sections were stored at −20 °C until further use.

### In vitro autoradiography

Radiotracer binding was evaluated by in vitro autoradiography with sections of human carotid plaques (20 μm). Cryosections were thawed at rt for 30 min and preincubated for 10 min on ice in HEPES buffer (50 mM HEPES, 5 mM MgCl_2_, 125 mM NaCl, 1 mM CaCl_2_, pH 7.4) supplemented with 0.5 % milk powder and 40 μM gammanorm (Octapharma AG, Lachen, Switzerland). Gammanorm contains human immunoglobulins that block unspecific binding of belatacept to ubiquitous Fc receptors. Tissues were incubated with 17 nM ^111^In-DOTA-belatacept (specific activity 36.6 GBq/μM) diluted in the above-specified buffer (~100 μL) for 1 h on ice. For blockade conditions, additionally 10 μM unlabeled belatacept was added to the tracer solution. The sections were washed with HEPES buffer containing milk powder and gammanorm (5 min, 4 °C), 3× HEPES buffer (5 min, 4 °C), and finally 2× distilled water (10 s, 4 °C) and were dried at rt. Standards of 1 μL tracer solution dried on 0.87 cm^2^ filter papers were prepared as positive controls. The sections and the standards were exposed to a super-resolution imager plate (PerkinElmer) for 30 min and 14 h. The plate was scanned by a Cyclone^®^ Plus Storage Phosphor System (PerkinElmer), and data was analyzed with the software OptiQuant (5.0, PerkinElmer). Specific binding was calculated from the difference between the integrated radioactivity signals per area (DLU/mm^2^) under baseline and blocking conditions, divided by the value under baseline conditions. Hematoxylin and eosin (HE) staining was performed with all sections to investigate tissue morphology and score each individual plaque specimen used in autoradiography according to Additional file 1: Table S3. A direct comparison with immunohistochemistry for CD80 and/or CD86 as performed in our previous study [7] was not performed as both ^111^In-DOTA-belatacept autoradiography with paraffin-embedded tissues and immunohistochemistry with cryosections were not successful. In addition, the poor resolution of the ^111^In autoradiography did not allow the microscopic localization of tracer accumulation in the sections. In total, 37 carotid specimens were used for autoradiography experiments. The distribution of the plaques per classification category and the individual details on patients and tissues are shown in Additional file 1: Tables S2 and S4.

### Statistics

Statistical data analysis was performed with GraphPad Prism (GraphPad, La Jolla, CA, USA) or Microsoft Excel. To assess the intergroup difference of two groups, a two-tailed unpaired Student’s *t* test was performed. For more than two groups, data was analyzed with a one-way ANOVA with a Tukey’s multicomparison test. A *p* value <0.05 was considered significant.

## Results

### Conjugation, radiolabeling, and quality control of ^111^In-DOTA-belatacept

The bifunctional chelating agent *p*-SCN-Bn-DOTA was successfully conjugated to belatacept under aqueous conditions by reaction with lysine amino groups of the protein according to a published procedure [14]. A 20-fold excess of chelating agent resulted in an average DOTA/protein molar ratio of 1.9 as determined by LC-MS. The so prepared protein conjugate was labeled with indium-111 and purified by semi-preparative fast-protein liquid chromatography (FPLC). After purification, ^111^In-DOTA-belatacept was obtained in a radiochemical yield of 73–78 %, a radiochemical purity of >99 %, and a specific activity of 36–41 GBq/μmol (*n* = 3).

### Stability of ^111^In-DOTA-belatacept

The stability of ^111^In-DOTA-belatacept at 37 °C in PBS and human and murine plasma, respectively, was investigated by FPLC up to 72 h (Additional file 1: Figure S1). More than 75 % of radiolabeled product was stable after 72 h incubation. After 48 h, corresponding to the time of most in vivo experimentation, 96 % intact product was present in murine plasma. In general, ^111^In-DOTA-belatacept displayed better stability in plasma than in PBS.

### Binding of ^111^In-DOTA-belatacept to CD80/CD86-positive Raji cells

**Fig. 1:**
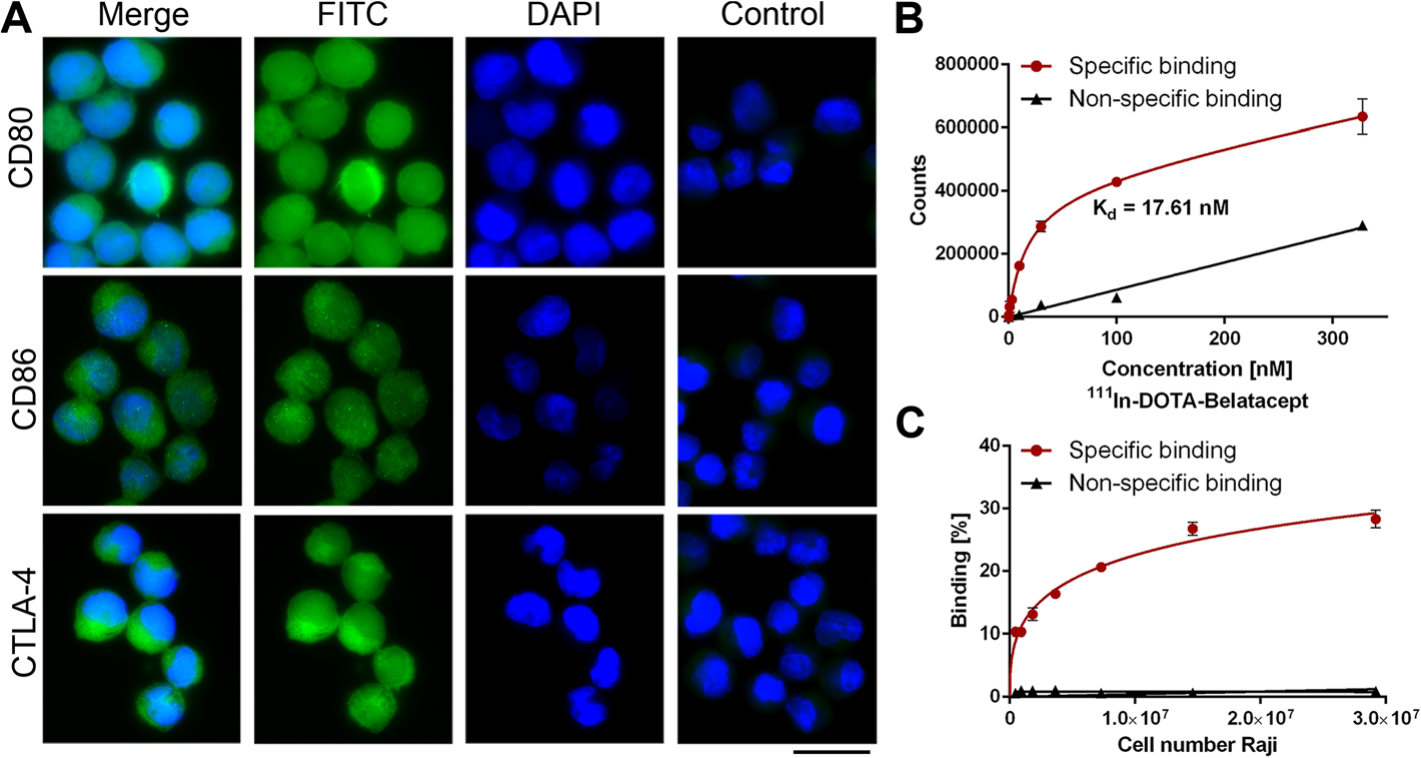
**a** Representative immunofluorescence microscopy images of Raji cells stained with DAPI (*blue*) and anti-CD80, anti-CD86, or anti-CTLA-4 antibody (*green*). The control samples were incubated with secondary antibody only. *Scale bar* 20 μm. **b** Saturation binding assay of ^111^In-DOTA-belatacept with Raji cells. Specific binding was calculated by subtracting the non-specific binding (^111^In-DOTA-belatacept and excess unlabeled belatacept) from the total binding (^111^In-DOTA-belatacept only). **c** Lindmo binding assay with a constant ^111^In-DOTA-belatacept concentration of 0.5 nM

Immunocytochemistry experiments confirmed the presence of CD80, CD86, and CTLA-4 on the surface and/or in the cytoplasm of Raji cells (Fig. 1a), in agreement with previous studies [17, 18]. A dissociation constant (*K*_d_) of 17.6 nM for ^111^In-DOTA-belatacept was determined in a saturation binding assay with CD80/CD86-positive Raji cells (Fig. 1b). The binding potential of ^111^In-DOTA-belatacept to Raji and control NCI-H69 cells was in addition evaluated in Lindmo assays. In Raji cells, the immunoreactivity of ^111^In-DOTA-belatacept was 28.3 % and binding was considered specific as incubation with 0.1 mM unlabeled belatacept resulted in a reduced radiotracer binding (Fig. 1c). Binding to NCI-H69 cells was below 5 % and non-specific (data not shown), in agreement with the absence of CD80/CD86 [7].

### Accumulation of ^111^In-DOTA-belatacept in CD80/CD86-positive Raji xenografts in vivo

The in vivo distribution of ^111^In-DOTA-belatacept and its accumulation in CD80/CD86-positive Raji and control NCI-H69 xenografts was evaluated in CD1 nu/nu mice. We have recently shown high expression of CD80 and CD86 mRNA and protein in Raji xenografts and negligible levels in NCI-H69 xenografts, both grown under identical experimental conditions as in this study [7]. In addition, immunofluorescence microscopy performed for CD86 confirmed its high level in Raji and low level or absence in NCI-H69 xenografts (Additional file 1: Figure S2).

**Fig. 2:**
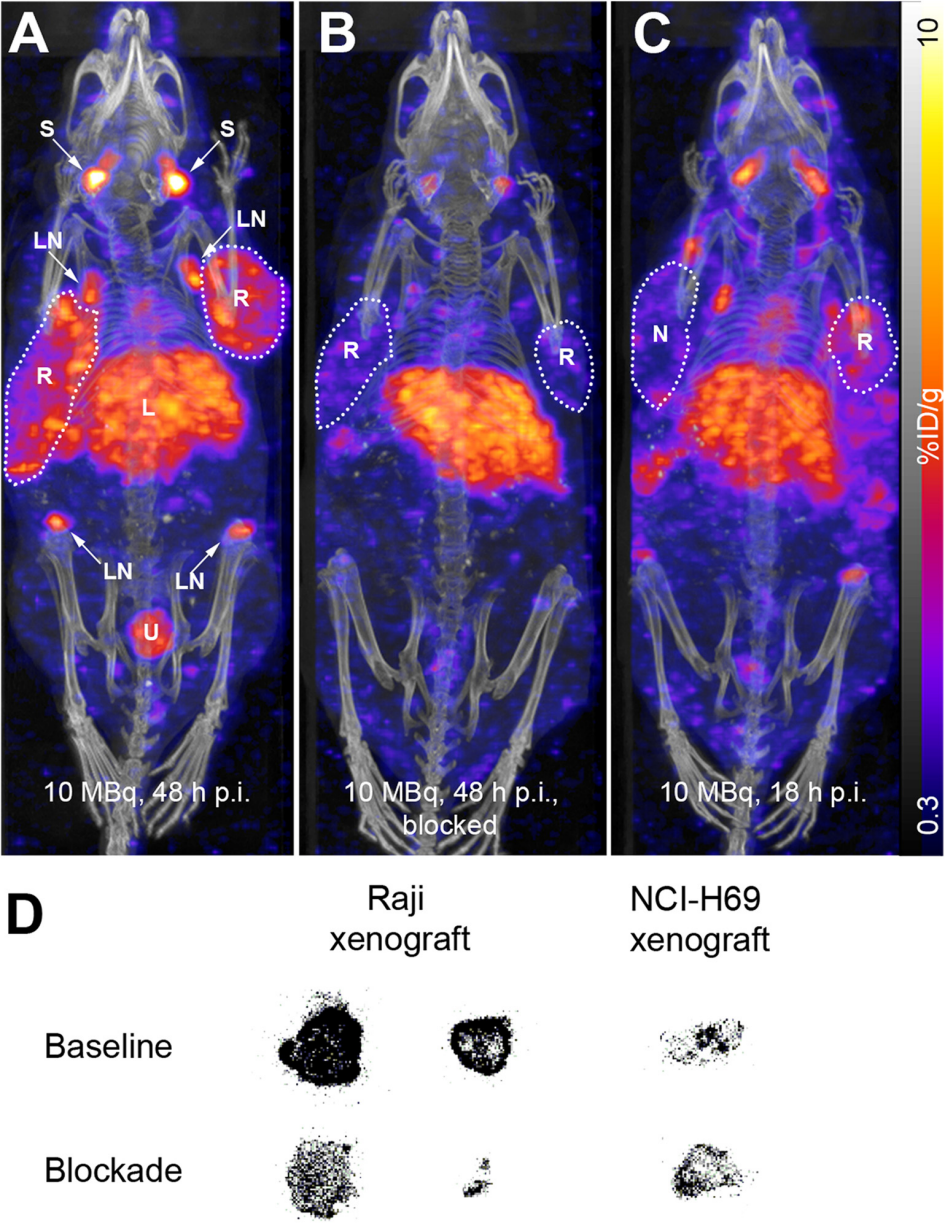
**a**–**c** In vivo SPECT/CT images acquired 48 h (**a**, **b**) or 18 h (**c**) after i.v. injection of ~10 MBq ^111^In-DOTA-belatacept (25 μg) in CD1 nu/nu mice bearing Raji (*R*, *encircled*) and a NCI-H69 xenograft (*N*, *encircled*) on the shoulders, as indicated. **b** Blocking with 500 μg unlabeled belatacept. *L* liver, *LN* lymph nodes, *S* salivary glands, *U* urinary bladder. *Colors*, SPECT signal (estimated % ID/g) according to scale bar; *gray*, computed tomography (CT). **d** Representative ex vivo autoradiograms of Raji and NCI-H69 xenografts dissected 48 h p.i. of ~10 MBq ^111^In-DOTA-belatacept (exposed on identical plate)

SPECT/CT scans were acquired 48 h (Fig. 2a, b), 18 h (Fig. 2c), or 2 h (Additional file 1: Figure S2) after tracer injection. Under baseline conditions, ^111^In-DOTA-belatacept accumulated in the Raji xenografts, the axial and inguinal lymph nodes, the salivary glands, and the liver (Fig. 2a). The highest accumulation in Raji xenografts was achieved in the scan 48 h after tracer injection. By co-injection of the radiotracer and an excess of unlabeled belatacept, radiotracer accumulation was reduced in the Raji xenografts, the lymph nodes, and the salivary glands while the radioactivity was still high in the liver (Fig. 2b). The mouse scanned 18 h post injection (p.i.) in Fig. 2c carried both a Raji and an NCI-H69 xenograft for direct comparison. As expected, tracer uptake was higher in the Raji than the NCI-H69 xenograft. Neither the Raji nor the NCI-H69 xenograft was visible in the scan 2 h after injection while blood radioactivity was still high as seen from the high signal in the carotid arteries and the heart (Additional file 1: Figure S2).

Ex vivo autoradiography analysis of CD80/CD86-positive Raji and control NCI-H69 xenografts were in agreement with the SPECT/CT data. In autoradiograms, we observed an overall higher radioactivity signal in Raji xenografts under baseline than under blockade conditions with a focal distribution pattern (Fig. 2d). The control NCI-H69 xenograft displayed a low radiotracer accumulation under baseline and blockade conditions indicating a non-specific uptake of ^111^In-DOTA-belatacept that was comparable to the signals observed in Raji xenografts under blockade conditions (Fig. 2d).

Quantitative radiotracer distribution in xenograft-bearing CD1 nu/nu mice was evaluated in an ex vivo biodistribution experiment with three animals each, 48 h after injection of ^111^In-DOTA-belatacept (baseline) or ^111^In-DOTA-belatacept together with unlabeled belatacept (blockade). Under baseline conditions, the highest percentage of injected dose per g tissue (% ID/g) was found in the spleen, the axial and inguinal lymph nodes, the liver, and the salivary glands, followed by the Raji xenografts and the blood (Table 1). Radiotracer accumulation was significantly higher in Raji than in NCI-H69 xenografts. Under blockade conditions, a significant reduction of radiotracer accumulation was observed for the Raji xenografts (*p* = 0.0057, uncorrected 2-tailed *t* test) and salivary glands (*p* = 0.0019).

**Table 1:**
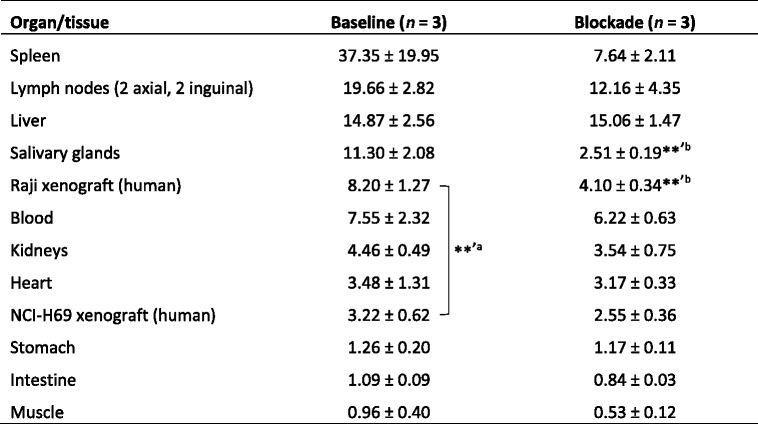
Biodistribution data (% ID/g tissue) of baseline (*n* = 3) and blockade (*n* = 3) CD1 nu/nu mice with subcutaneous Raji and NCI-H69 xenografts

**p < 0.01, uncorrected 2-tailed *t* test

^a^Comparing tissues under baseline conditions

^b^Comparing a particular tissue under baseline and blocking conditions

### Accumulation of ^111^In-DOTA-belatacept in murine atherosclerotic plaques

ApoE KO mice on regular diet develop atherosclerotic plaques in the aortic arch and the descending aorta. To enhance and accelerate plaque formation, ApoE KO mice were fed a high-fat diet. In two of these mice, plaques were further provoked in the carotid arteries by shear stress-modifying implants (cuffs; Meletta et al., manuscript submitted). Radiotracer accumulation in the atherosclerotic plaques was evaluated by ex vivo SPECT/CT imaging to allow an accurate localization of the SPECT signal. Tissues were dissected 48 h after injection of ^111^In-DOTA-belatacept.

**Fig. 3:**
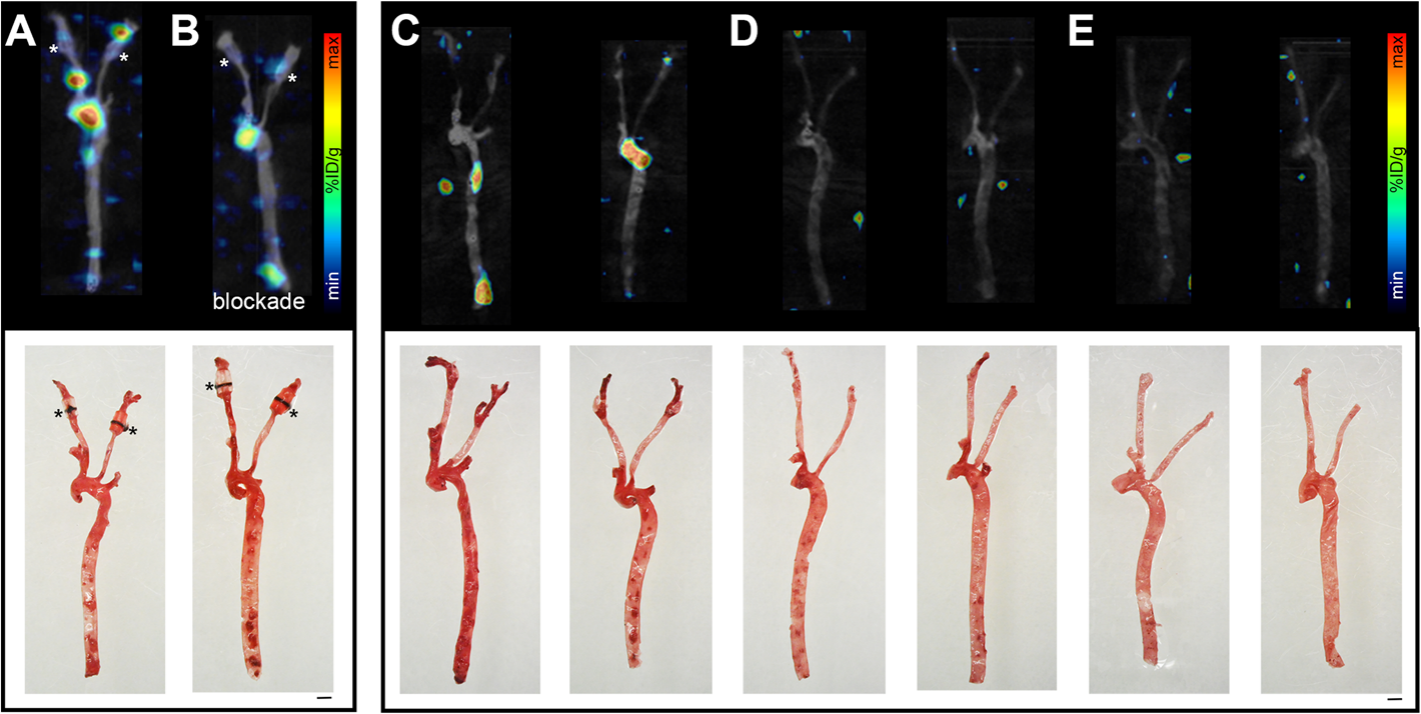
Ex vivo SPECT/CT images (*upper row*) and the corresponding oil red o staining (*bottom row*) of the aorta and carotids of ApoE KO-cuff mice fed a high-fat diet (**a**, **b**), ApoE KO animals fed a high-fat diet (**c**), ApoE KO animals fed a normal diet (**d**), and C57BL/6 mice fed a normal diet (**e**). Animals were injected intravenously with ~10 MBq ^111^In-DOTA-belatacept and sacrificed 48 h p.i. **b** Blockade with co-injection of an excess of unlabeled belatacept. *Color scales* for relative % ID/g. *Scale bars* 1 mm. ApoE KO-cuff mice (**a**, **b**) had flow-altering devices implanted around their carotids visible as transparent or reddish material fixed with a black suture (*asterisks*). **a** and **b** were directly compared in one scan, **c**, **d,** and **e** in a second scan (color scales may differ between the two panels)

In the implant-bearing animals, ^111^In-DOTA-belatacept accumulated in defined regions of the carotids besides the aortic arch and the descending aorta (Fig. 3a). The radiotracer signal co-localized with the lipid staining for atherosclerotic plaques; however, not all lipid-positive regions were positive in SPECT scans. Radiotracer accumulation was reduced under blockade conditions with an excess of unlabeled belatacept (Fig. 3b). Plaque burden was comparable in the baseline and the blockade tissues. Quantification of the SPECT signal revealed a higher radiotracer accumulation in the baseline than the blockade aorta and carotids. Ratios of maximal radioactivity between plaques and background (outside tissue) were 3.5 and 2.3 under baseline and blocking conditions, respectively, with a direct ratio of 1.5 between plaques baseline and blocked signal. In vivo SPECT/CT images of these mice, recorded before euthanasia, are provided in Additional file 1: Figures S3 and S4. Tracer accumulation was observed in the baseline animal upstream of the right implant and where the aortic arch is expected; however, high radioactivity in the adjacent tissues challenged the interpretation of these images.

In the two ApoE KO animals without a cuff, fed a high-fat diet, radiotracer accumulation was detected in the aortic arch and the descending aorta (Fig. 3c). Vascular segments with high radiotracer binding were positive by lipid staining, while additional large lipid-rich vascular areas were not identified by SPECT. Under a standard rodent diet, no ^111^In-DOTA-belatacept accumulation was observed in ApoE KO (Fig. 3d) and C57BL/6 mice (Fig. 3e). Oil red o staining revealed for the ApoE KO animals under normal diet a small lipid accumulation limited to the aortic arch, whereas in C57BL/6 animals, the lipid staining was negative.

### Accumulation of ^111^In-DOTA-belatacept in human carotid plaques

After the encouraging results with the mouse models of atherosclerosis, we investigated whether ^111^In-DOTA-belatacept accumulates in human atherosclerotic plaques and whether the accumulation correlates with characteristics of plaque vulnerability. Radiotracer binding to human carotid plaques was evaluated by in vitro autoradiography. After the autoradiography experiments, plaques were classified by histological analysis according to the following equally weighted criteria: (i) size of the lipid/necrotic core, (ii) number of immune cells (foam cells and macrophages), (iii) cap thickness, and (iv) cap rupture (Additional file 1: Table S3). The scale of total scores ranged from 0 to 8. In total, 37 plaque samples were included in this study with a heterogeneous distribution on the scoring scale and the majority of samples displaying a score of 0 to 2 (Additional file 1: Tables S2 and S4).

**Fig. 4:**
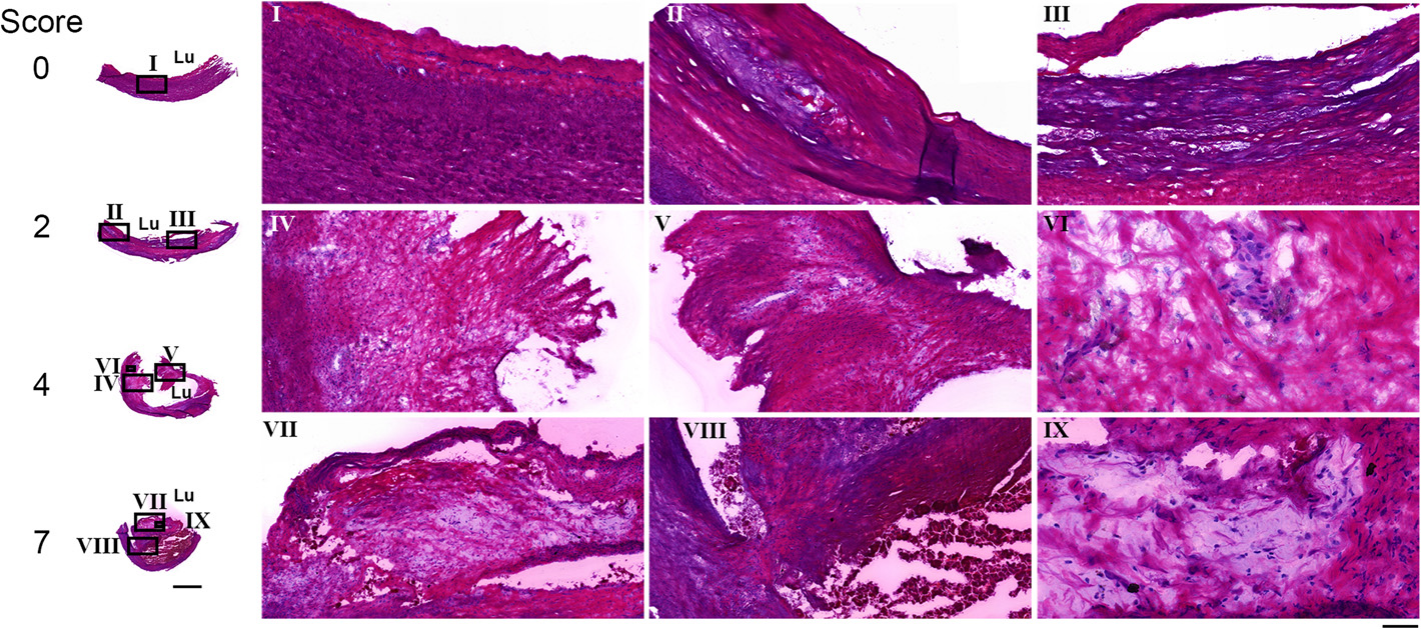
Hematoxylin/eosin staining of the plaque sections corresponding to the autoradiograms in Fig. 5. Higher-magnification images show the intact endothelium (*I*), endothelial thickening (*II*, *III*), a homogenous and thick fibrous cap (*IV*, *V*), foam cells (*VI*), a heterogeneous fibrous cap (*VII*), a thin fibrous cap (*VIII*), and immune cells (*IX*). *Scale bar* lower magnification 2 mm, higher magnification 200 μm, and VI/IX 50 μm. *Lu* lumen

Figure 4 shows the HE staining of four plaque specimens classified with a score of 0, 2, 4, and 7, respectively. The sample with a score of 0 displayed an intact and structured endothelium with no enclosed lipids or immune cells (Fig. 4, I). A small focal accumulation of lipids and immune cells was observed in the sample classified with a score of 2 (Fig. 4, II, III). The sample classified with a score of 4 featured a large lipid and necrotic core covered by a thick fibrous cap (Fig. 4, IV, V). The plaque was focally infiltrated by numerous macrophages and clusters of foam cells (Fig. 4, VI). The sample with the highest score consisted of a large lipid and necrotic core and displayed a heterogeneous plaque composition (Fig. 4, VII) and the presence of many immune cells (Fig. 4, IX). The representative and the minimum cap thickness were <500 and <200 μm, respectively (Fig. 4, VII, VIII).

**Fig. 5:**
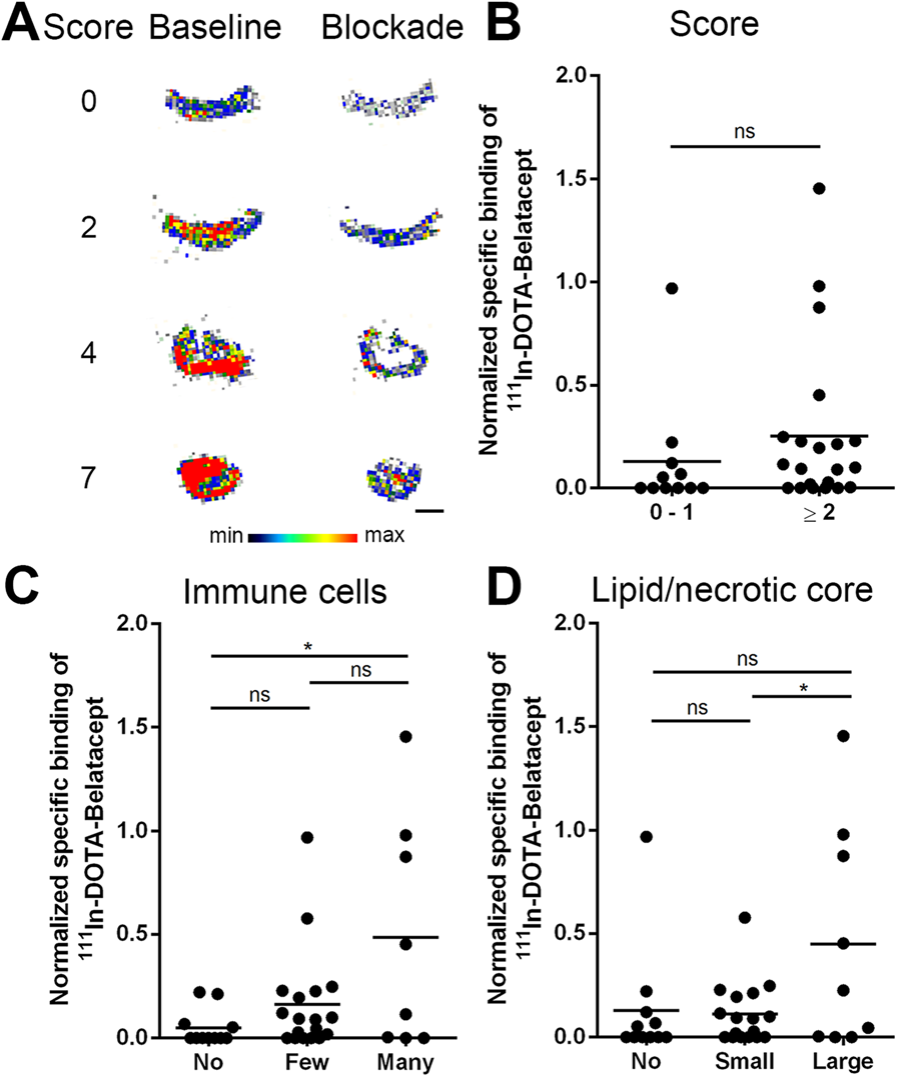
**a** In vitro autoradiograms of human carotid plaques with different scores under baseline (^111^In-DOTA-belatacept) and blockade condition (^111^In-DOTA-belatacept with excess unlabeled belatacept). *Scale bar* 3 mm. **b**–**d** Relative specific binding of ^111^In-DOTA-belatacept determined by in vitro autoradiography. Samples were classified according to their score (0–1: *n* = 11; ≥2: *n* = 21); no (*n* = 11), few (*n* = 18, <50 cells), or many (*n* = 8, ≥50 cells) immune cells (macrophages and foam cells); no (*n* = 11), small (*n* = 17, <50 % plaque thickness, <25 % total cross-sectional area), or large (*n* = 9, ≥50 % plaque thickness, ≥25 % total cross-sectional area) lipid/necrotic core. *ns* non-significant, *p < 0.05

In the in vitro autoradiography experiment, ^111^In-DOTA-belatacept bound to human carotid plaques and the radiotracer binding was reduced under blockade conditions with excess of unlabeled belatacept (Fig. 5a, corresponding plaque samples to Fig. 4). Average radiotracer specific binding to samples with a score ≥2 was higher than to samples with a score of 0–1, though not with statistical significance (Fig. 5b). However, the specific binding of ^111^In-DOTA-belatacept correlated significantly with the score for immune cell infiltration. Moreover, a significantly higher specific radiotracer binding was observed to plaques featuring a large lipid and necrotic core than to plaques with a small core (Fig. 5d). Radioactivity accumulation and cap thickness did not correlate, and the correlation between relative specific radioactivity and total score was weak with a Pearson’s *r*^2^ of 0.275 (details of the individual samples are shown in Additional file 1: Table S2). In these experiments, unspecific binding to Fc receptors was blocked by addition of human gamma globulin to the autoradiography buffer. The respective signal reduction was 10.9 ± 2.6 % (*n* = 3).

## Discussion

In this proof-of-concept study, we successfully established a radiolabeling procedure for the CD80/CD86-targeting fusion protein belatacept with indium-111 using *p*-SCN-Bn-DOTA as bifunctional chelating agent. After purification, the product was obtained in high radiochemical yield and purity in a fast and robust labeling reaction. Under the applied conditions, approximately two DOTA chelators were coupled to belatacept which is the intended range to minimize undesired chelator-mediated effects on pharmacokinetics [19]. A high stability of the radiolabeled product was determined in PBS and plasma of murine and human origin, respectively, up to 72 h allowing in vivo investigations.

The radiolabeled product bound to human Raji cells with a nanomolar *K*_d_ value. Efforts to assess the binding affinity to mouse recombinant CD80 by differential scanning fluorimetry were not successful due to similar melting temperatures *T*_m_ of the recombinant CD80 and belatacept. Immune-reactive affinity assays were excluded due to interferences with the Fc portion of belatacept. However, the results of this study indicate that the radiolabeling modifications did not hamper high-affinity binding to human and murine CD80/CD86 in vitro and in vivo. The contribution of Fc receptor-mediated binding to the total binding of belatacept was negligible as concluded from in vitro autoradiography with human tissue and as expected from published data [13].

In vivo SPECT/CT scans and ex vivo autoradiography experiments indicated that ^111^In-DOTA-belatacept specifically accumulated in CD80/CD86-positive tissues. Besides Raji xenografts, strong and specific accumulation was observed in the lymph nodes. The lymph nodes exhibit a resident population of APCs capable of expressing CD80 and CD86 in particular during the process of antigen presentation. Moreover, biodistribution studies revealed the highest radiotracer accumulation in the spleen, a peripheral lymphoid organ, and a high specific accumulation in the salivary glands of xenograft-bearing mice. This is consistent with the fact that APCs are constituents of human and murine salivary glands being involved in immune surveillance [20, 21]. The high non-specific hepatic radiotracer accumulation may be related to the IgG1 Fc part of the fusion protein [22].

The accumulation of radioactivity in the Raji xenografts increased from 2 to 18 to 48 h p.i. This is in agreement with the relatively long half-life in the blood of IgG1 Fc fusion proteins. The half-life of the closely related abatacept in mice was ~90 h after i.v. injection [23]. Indium-111 is thus a good choice for the labeling. Its long physical half-life of 67 h allows scans up to several days after tracer injection when a significant portion of ^111^In-DOTA-belatacept is cleared from the blood.

The high and specific accumulation of ^111^In-DOTA-belatacept in the lymph nodes of the xenograft-bearing animals seen in SPECT and confirmed in the biodistribution experiments looks promising towards the imaging of small CD80/CD86-rich structures. We, therefore, proceeded to an animal model of atherosclerosis where lesions are of similar or smaller dimension. However, owing to the proximity of atherosclerosis-prone areas in the aorta and the carotids to other tissues with radiotracer accumulation, e.g. the liver, the salivary glands, and the thymus, SPECT/CT scans of aorta and carotids were performed ex vivo. Furthermore, perfusion of the excised tissue allowed to exclude any blood-related tracer radioactivity, considering the long residence time of ^111^In-DOTA-belatacept in the blood (still 7.6 % ID/g blood 48 h after injection). ^111^In-DOTA-belatacept accumulated in lipid-rich atherosclerotic plaques, and tracer accumulation was higher under baseline than blockade condition. Moreover, ^111^In-DOTA-belatacept accumulation in the aortas of ApoE KO animals was dependent on an atherosclerosis-promoting diet. However, several lipid-rich regions were not positive in SPECT. We can only speculate on the reasons. SPECT imaging with ^111^In-DOTA-belatacept may not have been sensitive enough to visualize all lesions, depending on their size and tracer uptake. The signal was close to the detection limit in the ex vivo scans. Alternatively and in addition, not all lipid-rich regions may be invaded by high numbers of CD80/CD86-positive cells. As discussed under limitations, we did not further investigate these vessel walls by immunohistochemistry. However, we found that the descending aortas of ApoE KO mice fed a high-fat diet contained significantly higher mRNA levels of CD80 than the descending aortas of wild type mice under normal diet (Meletta et al., manuscript submitted). In agreement with our findings, the correlation between characteristics of plaque vulnerability and the lipid content of the lesions may be weak as derived from intravascular ultrasound measurements [24].

In human carotid tissue samples, ^111^In-DOTA-belatacept binding was associated with the infiltration of immune cells. It can be assumed that the majority of inflammatory cells within a plaque are APCs [25]. The increasing number of macrophages and mature DCs in advanced atherosclerotic lesions might promote the formation of a necrotic core due to a loss of efferocytosis activity, an intensification of the inflammatory response, and degradation of matrix components [1, 26, 27]. This is in agreement with the observed increase in ^111^In-DOTA-belatacept uptake in lesions with a large lipid/necrotic core. Our results suggest that ^111^In-DOTA-belatacept accumulates specifically in atherosclerotic plaques, depending on their degree of inflammation and vulnerability.

Limiting factors are related to the small size of atherosclerotic plaques both in preclinical models and in humans. High specific accumulation of radioactivity and low spill-over from other tissues are required for the detection of lesions in the millimeter or submillimeter range [28]. The long biological half-life of ^111^In-DOTA-belatacept is unfavorable in this respect, as it contributes to significant unspecific tissue radioactivity as observed in the in vivo scans. Imaging vulnerable plaques in coronary arteries would be even more challenging. According to the biodistribution results, radioactivity in myocardium was still relatively high 48 h p.i., although unspecific and lower than in the blood. This relatively high background level in the heart together with the respiratory and myocardium motion would hamper detection of atherosclerotic lesions in coronary arteries under these experimental conditions. A tracer with a faster clearance from the blood and CD80/CD86-negative tissues in combination with motion correction from simultaneously acquired magnetic resonance imaging (MRI) may improve the contrast between atherosclerotic lesions and myocardium in the future.

Further limitations of our study are the low number of animals used for in vivo and ex vivo imaging and the lack of control animals to study lymph node accumulation of ^111^In-DOTA-belatacept in the absence of xenografts or atherosclerosis. In addition, belatacept binds with similar affinity to CD80 and CD86, which may reduce selectivity for a particular type of cell activation as compared to a CD80- or CD86-selective tracer. Due to technical limitations with available antibodies, we were not able to counterstain the atherosclerotic tissues from SPECT and autoradiography for CD80 and CD86, the proteins that we found upregulated in human vulnerable atherosclerotic plaques [7]. Finally, a fusion protein would not fulfill all requirements for clinical application as a PET imaging agent primary due to its prolonged biological half-life of 8–9 days in humans in the case of belatacept and concerns with respect to immunogenicity of proteins [29].

Future studies have to focus on CD80- or CD86-selective small molecules and truncated versions of belatacept as well as abatacept, which has a higher affinity and selectivity for CD80 than CD86 in mouse [13]. In this respect, ^111^In-DOTA-belatacept could serve as a benchmarking tracer in the preclinical development of novel CD80/CD86 imaging agents.

## Conclusions

CD80 and CD86 expressed by activated APCs are promising imaging targets in atherosclerosis. In this context, ^111^In-DOTA-belatacept could serve as a research tool to study the approach of targeting CD80/CD86-rich APCs in atherosclerosis and possibly other inflammation-related diseases such as cancer, autoimmune disease, and rejection after organ transplantation. A highly expedient characteristic of belatacept is its immunoreactivity with both murine and human CD80/CD86 allowing the translation between species.

## Additional file

Additional file 1: **Supplementary information.** This file contains supplementary **Tables 1–4.** and **Figures 1–4.** and supplementary material and methods. (PDF 1198 kb)
