# Supplementary material for: Preclinical imaging of the co-stimulatory molecules CD80 and CD86 with indium-111-labeled belatacept in atherosclerosis
Source: EJNMMI Res. 2016 Jan 4;6:1. doi: 10.1186/s13550-015-0157-4 (PMC4700042; doi:10.1186/s13550-015-0157-4)
Supplement: Supplementary file 1 — Supplementary information. This file contains supplementary Tables 1–4. and Figures 1–4. and supplementary material and methods. (PDF 1198 kb) [file 13550_2015_157_MOESM1_ESM.pdf]

Supplementary Material

Preclinical imaging of the costimulatory molecules CD80 and CD86 with indium-111 labeled belatacept in atherosclerosis

Authors

Romana Meletta[1], Adrienne Müller Herde[1], Patrick Dennler[2], Eliane Fischer[2], Roger Schibli[1,2], Stefanie D. Krämer[1]

[1] Center for Radiopharmaceutical Sciences ETH-PSI-USZ, Institute of Pharmaceutical Sciences, Department of Chemistry and Applied Biosciences, ETH Zurich, Vladimir-Prelog-Weg 3/4, 8093 Zurich, Switzerland

[2] Center for Radiopharmaceutical Sciences ETH-PSI-USZ, Paul Scherrer Institute, OIPA10A, 5232 Villigen-PSI, Switzerland

Corresponding author

Stefanie D. Krämer

Center for Radiopharmaceutical Sciences ETH-PSI-USZ

Institute of Pharmaceutical Sciences

Department of Chemistry and Applied Biosciences

ETH Zurich

Vladimir-Prelog-Weg 4

CH-8093 Zurich, Switzerland

Tel.: +41 44 633 74 03

E-mail address: stefanie.kraemer@pharma.ethz.ch

Supplementary Tables

Supplementary Table 1 Assignment of the animals to experiments.

Animal ID	Strain	Xenografts or treatment	Baseline/ blockade	Experiments (Figures)
1	CD1 nu/nu	Raji (left/right)	Baseline	In vivo SPECT/CT (Fig. 2A)<br>Ex vivo autoradiography (Fig. 2D)
2	CD1 nu/nu	Raji (left/right)	Baseline	In vivo SPECT/CT<br>Ex vivo autoradiography
3	CD1 nu/nu	Raji (left/right)	Blockade	In vivo SPECT/CT (Fig. 2B)<br>Ex vivo autoradiography (Fig. 2D)
4	CD1 nu/nu	Raji (left/right)	Blockade	In vivo SPECT/CT<br>Ex vivo autoradiography
5	CD1 nu/nu	NCI-H69 (left/right)	Blockade	In vivo SPECT/CT<br>Ex vivo autoradiography (Fig. 2D)
6	CD1 nu/nu	NCI-H69 (left/right)		Not used, no tracer left
7	CD1 nu/nu	Raji (right), NCI-H69 (left)	Baseline	In vivo SPECT/CT (Suppl. Fig. 2)<br>Ex vivo biodistribution (dissection)
8	CD1 nu/nu	Raji (right), NCI-H69 (left)	Baseline	In vivo SPECT/CT<br>Ex vivo autoradiography (Fig. 2D)<br>Ex vivo biodistribution (dissection)
9	CD1 nu/nu	Raji (right), NCI-H69 (left)	Baseline	In vivo SPECT/CT (Fig. 2C)<br>Ex vivo biodistribution (dissection)
10	CD1 nu/nu	Raji (right), NCI-H69 (left)	Blockade	In vivo SPECT/CT<br>Ex vivo biodistribution (dissection)
11	CD1 nu/nu	Raji (right), NCI-H69 (left)	Blockade	Ex vivo biodistribution (dissection)
12	CD1 nu/nu	Raji (right), NCI-H69 (left)	Blockade	Ex vivo biodistribution (dissection)
13	ApoE KO	High fat diet, cuffs	Baseline	In vivo SPECT/CT (Suppl. Fig. 3)<br>Ex vivo SPECT/CT (Fig. 3A)<br>Oil red o (Fig. 3A)
14	ApoE KO	High fat diet, cuffs	Blockade	In vivo SPECT/CT (Suppl. Fig. 4)<br>Ex vivo SPECT/CT (Fig. 3B)<br>Oil red o (Fig. 3B)
15	ApoE KO	High fat diet	Baseline	Ex vivo SPECT/CT (Fig. 3C)<br>Oil red o (Fig. 3C)
16	ApoE KO	High fat diet	Baseline	Ex vivo SPECT/CT (Fig. 3C)<br>Oil red o (Fig. 3C)
17	ApoE KO	Normal diet	Baseline	Ex vivo SPECT/CT (Fig. 3D)<br>Oil red o (Fig. 3D)
18	ApoE KO	Normal diet	Baseline	Ex vivo SPECT/CT (Fig. 3D)<br>Oil red o (Fig. 3D)
19	C57BL/6	Normal diet	Baseline	Ex vivo SPECT/CT (Fig. 3E)<br>Oil red o (Fig. 3E)
20	C57BL/6	Normal diet	Baseline	Ex vivo SPECT/CT (Fig. 3E)<br>Oil red o (Fig. 3E)

Supplementary Table 2 Details of the human tissue samples

Patient	Sample #	Location	Macro	Microscopic inspection						Sex	Age	Indication	Pharmacology, smoking	AR
				Plaque	Core	IC	Cap thinn.	Cap rupt.	Score					
1	1	ACE	v	yes	1	1	0	0	2	m	82	na	na	No sp. bi.
2	1	A. poplitea	s	no	0	0	0	0	0	m	62	na	na	No sp. bi.
3	1	ACI	v	yes	1	1	0	0	2	m	74	na	na	No sp. bi.
4	1	ACC	n	no	0	0	0	0	0	m	85	TIA	DM, AH, HC, St, BB, ACEI, PAI	No sp. bi.
5	1	ACE	s	yes	1	1	0	0	2	m	85	Stroke	AH, HC, Sm, PAI	No sp. bi.
6	1	ACI	s	yes	2	2	1	0	5	f	70	asymptomatic	AH, St, BB, CA, ACEI, PAI	No sp. bi.
7	1	ACI	v	yes	1	1	na	na	na	m	74	symptomatic (no TIA, no stroke)	na	No sp. bi.
8	1	na	s	no	0	0	0	0	0	na	na	na	na	No sp. bi.
9	1	AC, na	v	yes	1	1	na	na	na	f	76	na	CA, PAI	No sp. bi.
10	1	ACE	s	no	0	0	0	0	0	m	76	na	DM, AH, Sm, St, AC, CA, ARA, PAI	No sp. bi.
10	2	ACE	s	no	0	0	0	0	0	m	76	na	DM, AH, Sm, St, AC, CA, ARA, PAI	No sp. bi.
11	1	ACE	v	yes	2	2	na	na	na	m	74	na	na	No sp. bi.
12	1	ACC	s	no	0	0	0	0	0	f	85	na	na	No sp. bi.
12	2	ACE	s	yes	1	0	1	0	2	f	85	na	na	No sp. bi.
7	2	ACI	v	yes	2	1	na	na	na	m	74	symptomatic (no TIA, no stroke)	na	0.004
13	1	ACE	v	yes	1	2	1	0	5	m	66	na	na	0.019
14	1	ACI	v	yes	1	1	0	0	2	m	74	symptomatic (no TIA, no stroke)	AH, HC, CA, PAI	0.029
11	2	ACI	v	yes	2	1	na	na	na	m	74	na	na	0.045
15	1	na	s	no	0	0	0	0	0	na	67	na	na	0.053
16	1	na	s	no	0	0	0	0	0	na	na	na	na	0.068
5	2	ACI	v	yes	1	1	0	0	2	m	85	Stroke	AH, HC, Sm, PAI	0.09
17	1	ACC	s	yes	1	1	0	0	2	m	71	symptomatic (no TIA, no stroke)	DM, AH, HC, St, BB, CA, ACEI, PAI	0.094
18	1	ACE	s	yes	1	1	0	0	1	m	68	asymptomatic	DM, AH, HC, Sm, St, ARA, PAI	0.1
17	2	ACI	s	yes	1	2	0	0	3	m	71	symptomatic (no TIA, no stroke)	DM, AH, HC, St, BB, CA, ACEI, PAI	0.115
19	1	ACE	s	yes	0	1	0	0	1	m	69	Stroke	AH, HC, St, BB, PAI	0.122
20	1	ACE	s	yes	1	1	0	0	2	m	82	Stroke	St, ACEI, PAI	0.196
10	3	ACI	v	yes	1	0	1	0	2	m	76	na	DM, AH, Sm, St, AC, CA, ARA, PAI	0.213
21	1	na	n	no	0	0	0	0	0	na	na	na	na	0.222
12	3	ACI	v	yes	2	1	0	0	3	f	85	na	na	0.227
22	1	ACE	s	yes	1	1	0	0	2	m	76	na	na	0.229
18	2	ACI / ACC	s	yes	1	1	0	0	2	m	68	asymptomatic	DM, AH, HC, Sm, St, ARA, PAI	0.249
23	1	ACI	n	yes	2	2	0	0	4	m	75	na	na	0.452
22	2	ACI	v	yes	1	1	na	na	na	m	76	na	na	0.577
11	3	ACC	v	yes	2	2	0	1	5	m	74	na	na	0.875
17	3	ACE	v	yes	0	1	0	0	1	m	71	symptomatic (no TIA, no stroke)	DM, AH, HC, St, BB, CA, ACEI, PAI	0.968
5	3	ACI	v	yes	2	2	2	1	7	m	85	Stroke	AH, HC, Sm, PAI	0.979
14	2	ACI	v	yes	2	2	0	0	4	m	74	symptomatic (no TIA, no stroke)	AH, HC, CA, PAI	1.454

Macro, macroscopic characterization by the surgeon; Microscopic inspection, see Supplementary Table 3 for details; IC, immune cells; thinn., thinning; rupt., rupture; AR, autoradiography relative specific signal shown in Figure 5; ACE, ACI, ACC, arteria carotis externa, interna, communis; v, s, vulnerable, stable plaque; n, normal tissue; na, not available; m, male; f, female; TIA, transient ischemic attack; DM, diabetes mellitus; AH, arterial hypertension; HC, hypercholesterolemia; Sm, smoking; St, statins; BB, beta blocker; AC, anticoaculang; CA, calcium antagonist; ACEI, angiotensin converting enzyme inhibitor; ARA, angiotensin II receptor antagonist; PAI, platelet aggregation inhibitor; No sp. bi., no specific binding. Column-wise color coding, for patient numbers, increasing from white to grey; for scoring, age and AR, low (green), medium (yellow), high (red). Note, the investigated slice (microscopic inspection) may differ from the macroscopic gross characterization as tissues were in many cases heterogeneous.

Supplementary Table 3 Scoring system of histologically analyzed plaque features and the number of samples per classification category. The classification of the immune cells was performed according to the number of cells present relative to the overall plaque size. The term "immune cells" refers to macrophages and foam cells.

**Sample features**		**Score**	**Number of samples**
Lipid and necrotic core	No	0	11
	Small (< 50% plaque thickness, < 25% total cross-sectional area)	1	17
	Large (≥ 50% plaque thickness, ≥ 25% total cross-sectional area)	2	9
Immune cells	None	0	11
	Few (< 50 cells)	1	18
	Many (≥ 50 cells)	2	8
Minimum cap thickness	>200 µm	0	33
	<200 µm	1	4
Representative cap thickness	>500 µm	0	35
	<500 µm	1	2
Cap	Intact	0	35
	Possibly ruptured	1	2
	Definitely ruptured	2	0

Supplementary Table 4 Overview of all human endarterectomy samples and their respective score according to Supplementary Table 3. In some cases, no scoring of the sample was possible due to fragmentary plaque tissue e.g. no cap present hampering the morphological assessment of the plaques (indicated as n.d., non-determined).

**Overall score**	**Number of samples**
0	9
1	3
2	11
3	2
4	2
5	3
6	0
7	1
8	0
n.d.	6

Supplementary Figures

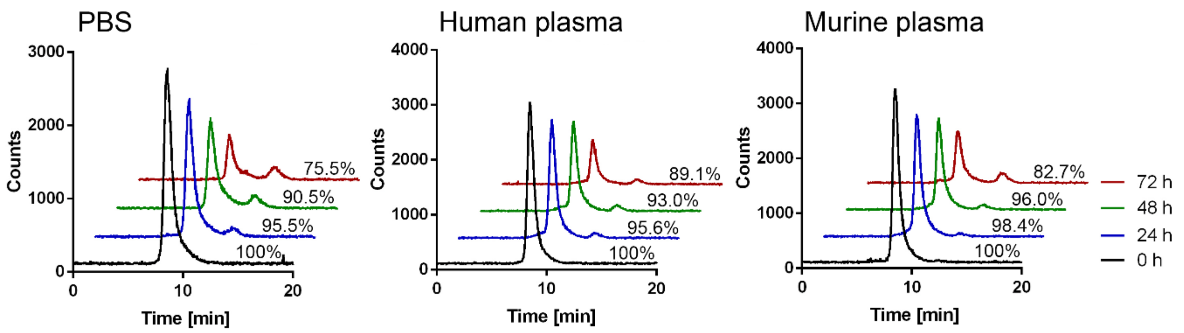

Supplementary Fig. 1 Stability of ^{111}In-DOTA-belatacept in PBS, human and murine plasma at 37°C analyzed by fast protein liquid chromatography (FPLC) up to 72 h. The percentage of intact ^{111}In-DOTA-belatacept at different analysis time points is indicated and was calculated based on the peak areas of the intact product (t_{R1} ~9 min) and degraded protein fragment(s) (t_{R2} ~12 min).

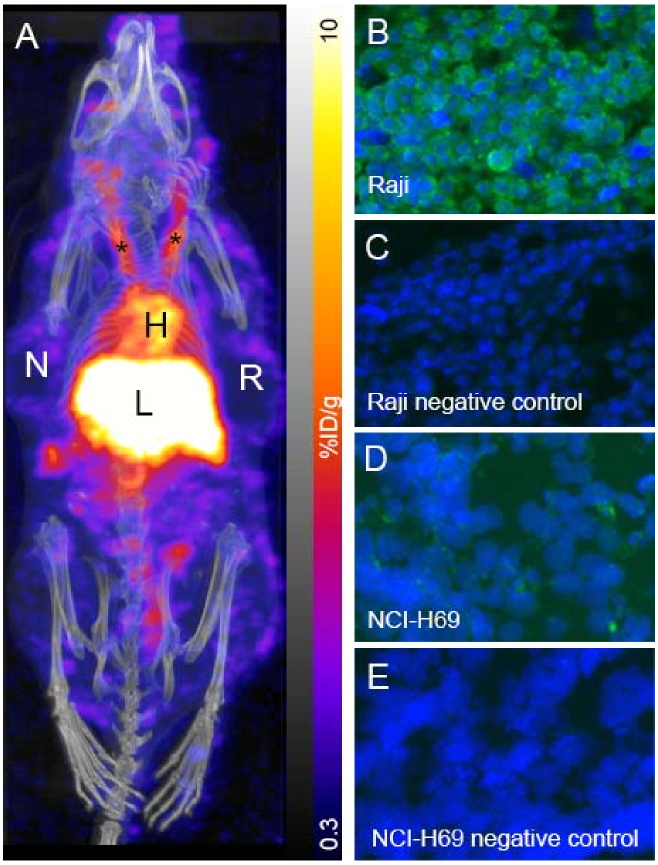

Supplementary Fig. 2 a) In vivo SPECT/CT image of a CD1 nu/nu mouse bearing a NCI-H69 (N) and a Raji (R) xenograft. The image was acquired 2 h p.i. of ~10 MBq ^{111}In-DOTA-belatacept (25 µg) via the tail vein. Color scale, SPECT % ID/g as indicated; grey, computed tomography (CT). The high tracer concentration in blood 2 h p.i. is evident from the high signal where the two Arteria carotis (indicated with asterisks) are located. L, liver; H, heart. **b**-**e**) Immunofluorescence micrographs of a Raji (**b**,**c**) and a NCI-H69 (**d**,**e**) xenograft grown under the same experimental conditions as for the SPECT imaging, labeled with 4',6-diamidino-2-phenylindole (DAPI, nuclei) and anti-CD86 antibody (**b**, **d**) or no primary antibody as negative controls (**c**, **e**).

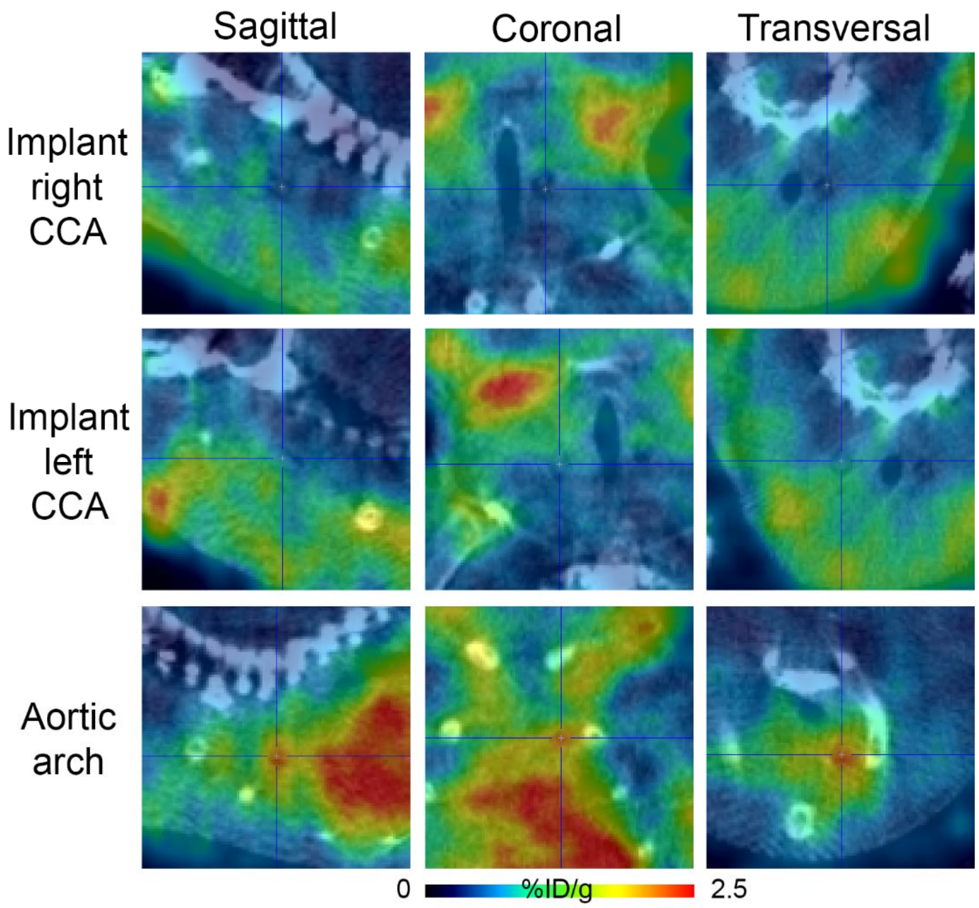

Supplementary Fig. 3 In vivo SPECT/CT images of an ApoE KO mouse injected with 10 MBq of ^{111}In-DOTA-belatacept. Images show the two flow-modifying implants placed around the right and left common carotid artery (CCA), visible as dark areas in CT, and the aortic arch. Color scale for estimated % ID/g as indicated.

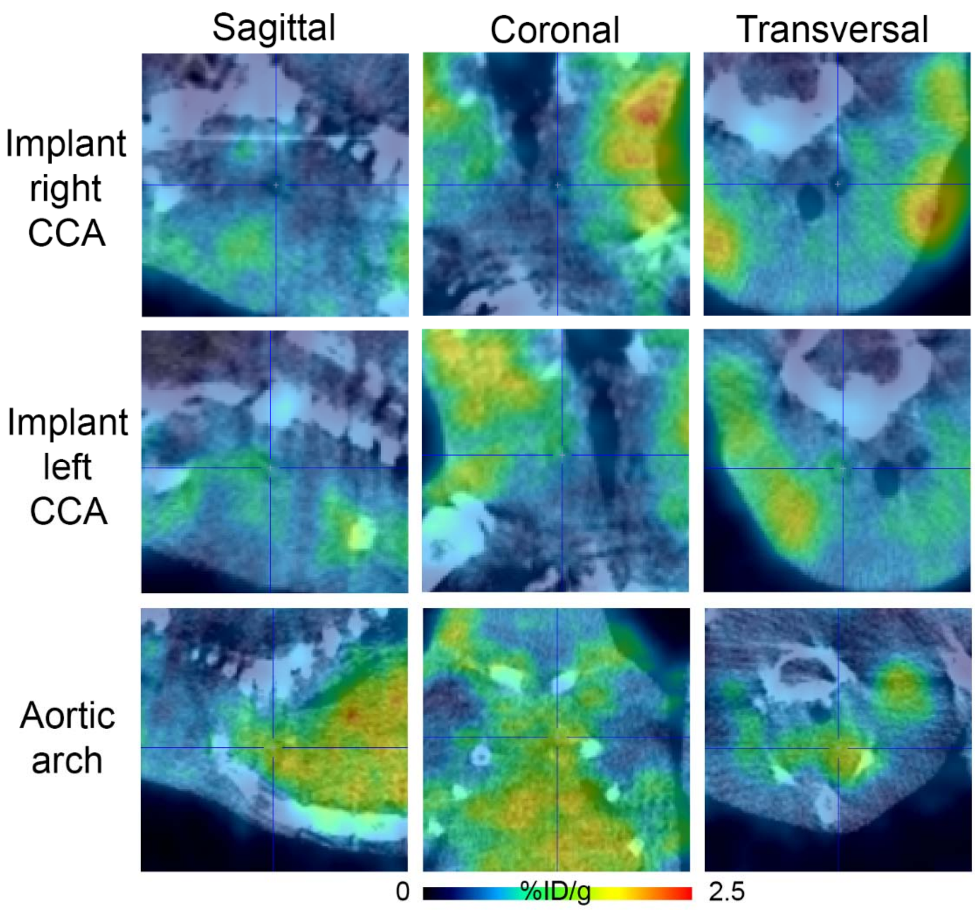

Supplementary Fig. 4 In vivo SPECT/CT images of an ApoE KO mouse injected with 10 MBq of ^{111}In-DOTA-belatacept and 500 µg unlabeled belatacept. Details and scale bar, see Supplementary Fig. 3

Supplementary Material and Methods

Conjugation, radiolabeling and quality control

Chemicals and solvents were purchased in trace analysis grade from Fluka or Sigma-Aldrich (Buchs, Switzerland) and were used without further purification. Briefly, conjugation of 92.3 µM belatacept was performed under metal-free conditions using a 20-fold molar excess of chelating agent. The reaction mixture was incubated overnight at 4°C yielding after purification in 1.4 mL protein conjugate solution with a concentration of 5.56 ± 0.02 mg/mL determined by a NanoDrop® ND-1000 spectrophotometer (Witec AG, Littau, Switzerland). Aliquots were stored at -20°C.

After radiolabeling (see main manuscript) the product (500 µL) was purified by FPLC on a Shimadzu SCL-10A VP system with a superose 12 column (GE Healthcare). Isocratic conditions with PBS (pH 7.4) as mobile phase and a flow rate of 0.5 mL/min were applied. Quality control of the purified product was performed under isocratic conditions (0.3 M NaCl, 0.05 M NaH_2PO_4, pH 6.2) with a TSKgel G3000SWxl column (7.8 mm x 30 cm, 5 µm; Tosoh Bioscience LLC). HPLC data were analyzed with the software RadioStar.

LC-MS analysis

LC-MS analysis was performed on a Waters LCT Premier mass spectrometer. Prior to analysis, conjugated belatacept was deglycosylated using a Protein Deglycosylation Mix (P6039, New England BioLabs Inc.). G7 reaction buffer (2.5 µL 10x concentrated) and 2.5 µL deglycosylation enzyme cocktail were added to 20 µL of 1.1 mg/mL belatacept in H_2O and the mixture was incubated at 37°C overnight. The deglycosylation mixture (20 µL) was mixed with 20 µL PBS (pH 7.4) and 2 µL 1 M DTT to reduce deglycosylated belatacept at 37°C for 30 min. Samples were chromatographed on an Aeris WIDEPORE XB-C18 column (3.6 µm, 100 mm x 2.1 mm; Phenomenex) heated to 80°C using the following gradient: 0 min to 3 min: 5% A, 90% B, 5% C; 3 min to 15 min: 5 to 75% A, 90 to 20% B, 5% C; 15 min to 20 min: 75 to 90% A, 20 to 5% B, 5% C (solvent A: acetonitrile + 0.1% formic acid, solvent B: water + 0.1% formic acid, solvent C: isopropanol) at a flow rate of 0.5 mL/min. The eluent was ionized using an electrospray source (ESI+). Data were collected with MassLynxV4.1 and deconvolution was performed using MaxEnt1. Payload ratios were calculated based on mass peak intensity.

Stability evaluation of ^{111}In-DOTA-belatacept

The in vitro stability of ^{111}In-DOTA-belatacept was evaluated in PBS (pH 7.4), human and murine (male NMRI nu/nu mice) blood plasma, respectively. Approximately 1.4 MBq radiotracer (20 µL, 76.5 nM) was added to 500 µL PBS or plasma and the samples were incubated at 37°C. Fifty-microliter samples were analyzed after 0, 24, 48 and 72 h by size-exclusion chromatography on a SCL-10A VP system

(Shimadzu) equipped with a UV/Vis and a radiodetector. Chromatographic analysis corresponded with the conditions applied for quality control of the radiolabed product.

Cell culture

Raji cells were maintained in Roswell Park Memorial Institute (RPMI) 1640 medium with GlutaMAX (Gibco, Life Technologies) supplemented with 10% heat-inactivated fetal calf serum and penicillin-streptomycin. NCI-H69 cells were cultured in RPMI 1640 medium with GlutaMAX and 25 mM HEPES (Gibco, Life Technologies) supplemented with 10% heat-inactivated fetal calf serum, penicillin-streptomycin and 1 mM sodium pyruvate. Cells were cultured in suspension at 37°C in a humidified atmosphere containing 5% CO_2. Routine culture treatment was performed twice a week.

Cell immunofluorescence microscopy

All incubation steps were performed at room temperature unless otherwise stated. Raji cells were seeded on 6-well tissue culture plates on the day of experiment and each well contained a disinfected coverslip. The cell culture medium was carefully removed and cells were fixed by incubation with 4% paraformaldehyde in PBS (pH 7.4) for 30 min. Thereafter, cells were permeabilized by incubation with methanol (10 min) and blocked with 2% bovine serum albumin (BSA) in TBS buffer (50 mM Tris, 150 mM NaCl, pH 7.4) for 30 min. The primary antibody was diluted to the appropriate concentration in 2% BSA/TBS and cells were incubated at 4°C overnight. The next day, cells were washed in TBS (3x, 10 min) and the secondary antibody was added in 2% BSA/TBS. The samples were incubated for 1 h and kept from that point on in dark. After washing in TBS (3x, 10 min), DAPI (1:5000) was added for 4 min to stain the nuclei of the cells. After three more washing steps with TBS (10 min each), the coverslips were mounted onto slides using ProLong® Gold antifade reagent (Life Technologies, Carlsbad, CA, USA) with cell-side facing down. For negative control, only secondary antibody was added to the cells. Slides were examined using an Axioskop 2 microscope equipped with 20/0.50 Plan-Neofluar, 40/1.3 oil Plan-Neofluar, and 63/1.25 oil Plan-Neofluar objectives and an AxioCam digital camera (Carl Zeiss AG, Feldbach, Switzerland). Images were acquired using the AxioVision software (Carl Zeiss AG).

Tissue immunofluorescence microscopy

Raji or NCI-H69 xenografts were dissected from euthanized mice and embedded in Tissue-Tek (Thermo Scientific). Frozen sections (7 µm) were cut on a cryostat, thaw-mounted onto coated glass slides (Superfrost plus, Menzel) and stored at −20°C until further use. Sections were fixed with ice cold methanol/aceton (1:1; v/v) for 5 min following a washing step with TBS for 5 min. The following blocking, incubation and washing procedures were performed under the same conditions as mentioned above for immunocytochemistry as well as the microscopic analysis.

Binding studies

Saturation binding and Lindmo assays with ^{111}In-DOTA-belatacept were performed with Raji and NCI-H69 cells. For the saturation binding assay, the cells (approx. 8.5 × 10^6 cells per tube) were washed twice with PBS and incubated for 90 min on ice with increasing concentrations of radiotracer (0.03 to 330 nM) in triplicates. The total volume per tube was adjusted with RPMI medium to 200 µL. Non-specific binding was determined by adding 100 µM unlabeled belatacept as displacer. After 2 h incubation, the cells were washed three times with 2 mL ice-cold PBS (pH 7.4) and centrifuged at 1260 x g for 5 min at 4°C (Sorvall RC-5C centrifuge, Thermo Fisher). Samples were analyzed in a γ-counter (Packard Cobra II Auto Gamma, PerkinElmer). The dissociation constant (K_d) was calculated by fitting the specific radiotracer binding with non-linear regression analysis assuming a 1:1 binding (GraphPad Prism Software). In the Lindmo assay a fixed amount of ^{111}In-DOTA-belatacept (0.5 nM) was incubated with a standard dilution (dilution factor 2) of Raji and NCI-H69 cells, respectively, in RPMI medium. The highest cell number for the Raji cells was approx. 2.9 × 10^7 cells/tube and for the NCI-H69 approx. 1.6 × 10^7 cells/tube. The total volume per tube was adjusted with RPMI medium to 200 µL. To assess the non-specific binding, 100 µM unlabeled belatacept was used as displacer. The samples were incubated for 2 h at 4°C, thereafter washed three times with 2 mL ice-cold PBS and centrifuged at 1260 x g for 5 min at 4°C. Samples were analyzed in a γ-counter (Packard Cobra II Auto Gamma, PerkinElmer).

Oil red o tissue staining

Lipid staining of the aorta and the carotids was accomplished with the dye oil red o at room temperature according to a previously published procedure [ref. S1]. In brief, the tissue was fixed in 4% PFA for 10 min, thereafter washed twice with PBS and preincubated in 60% 2-propanol for 5 min. Tissues were stained with 0.3% (w/v) oil red o (Sigma-Aldrich) in 60% 2-propanol for 30 min on a shaking platform. Finally, the tissue was washed under running tap water for 2 min. Images of the stained tissues were obtained with a Nikon SMZ1000 microscope equipped with a Nikon 1 J3 digital camera (Nikon, Melville, NY, USA).

Reference

[S1] Kim, S.H., Lee, E.S., Lee, J.Y., Lee, E.S., Lee, B.S., Park, J.E., and Moon, D.W. (2010). Multiplex coherent anti-stokes Raman spectroscopy images intact atheromatous lesions and concomitantly identifies distinct chemical profiles of atherosclerotic lipids. Circulation research *106*, 1332-1341.
